# Metabolomic and Proteomic Analyses of Persistent Valvular Atrial Fibrillation and Non-Valvular Atrial Fibrillation

**DOI:** 10.3389/fgene.2021.789485

**Published:** 2021-11-30

**Authors:** Bo Hu, Wen Ge, Yuliang Wang, Xiaobin Zhang, Tao Li, Hui Cui, Yongjun Qian, Yangyang Zhang, Zhi Li

**Affiliations:** ^1^ Department of Cardiology, Shanghai East Hospital, School of Medicine, Tongji University, Shanghai, China; ^2^ Department of Cardiothoracic Surgery, Shuguang Hospital, Affiliated to Shanghai University of TCM, Shanghai, China; ^3^ Department of Immunology, Nanjing Medical University, Nanjing, China; ^4^ Department of Cardiovascular Surgery, Shanghai Chest Hospital, Shanghai Jiao Tong University, Shanghai, China; ^5^ Department of Cardiovascular Surgery, National Clinical Research Center for Geriatric, West China Hospital, Sichuan University, Chengdu, China; ^6^ School of Life Science and Technology, Shanghai Tech University, Shanghai, China; ^7^ Department of Cardiovascular Surgery, Jiangsu Province Hospital, the First Affiliated Hospital of Nanjing Medical University, Nanjing, China

**Keywords:** metabolic, proteomic, valvular atrial fibrillation, non-valvular atrial fibrillation, persistent atrial fibrillation

## Abstract

Atrial fibrillation (AF) is an abnormal heart rhythm related to an increased risk of heart failure, dementia, and stroke. The distinction between valvular and non-valvular AF remains a debate. In this study, proteomics and metabolomics were integrated to describe the dysregulated metabolites and proteins of AF patients relative to sinus rhythm (SR) patients. Totally 47 up-regulated and 41 down-regulated proteins in valvular AF, and 59 up-regulated and 149 down-regulated proteins in non-valvular AF were recognized in comparison to SR patients. Moreover, 58 up-regulated and 49 significantly down-regulated metabolites in valvular AF, and 47 up-regulated and 122 down-regulated metabolites in persistent non-valvular AF patients were identified in comparison to SR patients. Based on analysis of differential levels of metabolites and proteins, 15 up-regulated and 22 down-regulated proteins, and 13 up-regulated and 122 down-regulated metabolites in persistent non-valvular AF were identified relative to valvular AF. KEGG pathway enrichment analysis showed the altered proteins and metabolites were significantly related to multiple metabolic pathways, such as Glycolysis/Gluconeogenesis. Interestingly, the enrichment pathways related to non-valvular AF were obviously different from those in valvular AF. For example, valvular AF was significantly related to Glycolysis/Gluconeogenesis, but non-valvular AF was more related to Citrate cycle (TCA cycle). Correlation analysis between the differentially expressed proteins and metabolites was also performed. Several hub proteins with metabolites were identified in valvular AF and non-valvular AF. For example, Taurine, D-Threitol, L-Rhamnose, and DL-lactate played crucial roles in valvular AF, while Glycerol-3-phosphate dehydrogenase, Inorganic pyrophosphatase 2, Hydroxymethylglutaryl-CoAlyase, and Deoxyuridine 5-triphosphate nucleotidohydrolase were crucial in non-valvular AF. Then two hub networks were recognized as potential biomarkers, which can effectively distinguish valvular AF and non-valvular persistent AF from SR samples, with areas under curve of 0.75 and 0.707, respectively. Hence, these metabolites and proteins can be used as potential clinical molecular markers to discriminate two types of AF from SR samples. In summary, this study provides novel insights to understanding the mechanisms of AF progression and identifying novel biomarkers for prognosis of non-valvular AF and valvular AF by using metabolomics and proteomics analyses.

## Introduction

Atrial fibrillation (AF) is the most common arrhythmia with an abnormal atrial rhythm, resulting in increasing risk of heart failure, and stroke (Zhou et al., 2019). AF attacks more than 30 million individuals in developed countries (Christophersen et al., 2016), and this number is expected to grow sharply in the next 20 years. However, the AF-associated atrial electrical, and structural remodeling mechanisms are more complicated and variable in patients compared with other types of arrhythmia. Various heart diseases may lead to atrial remodeling, resulting in the development of AF, whereas AF can also give rise to atrial remodeling due to the progression of others arrhythmia (Anne et al., 2005). The probable reason is that different risk factors contribute to adverse atrial remodeling in given individuals to induce AF. In clinical practice, AF can be divided into paroxysmal AF (up to 7 days with spontaneous termination), persistent AF (more than 7 days, needing medical or electrical cardioversion), long-standing persistent AF (sustaining at least 1 year) and permanent AF (long-term maintenance of sinus rhythm not an option) (Fauchier et al., 2015). Heart valve-related AF is also most common in patients with rheumatic mitral stenosis, and the non-valvular related AF refers to the unclear etiology with no valvular heart disease (Fauchier et al., 2015). Despite a large number of basic and clinical studies, little is known about the basic mechanical difference between valvular and non-valvular AF. In addition, identifying more reliable biomarkers for early diagnosis is urgent and can help understand the pathological mechanism of AF and provide new therapeutic targets.

Recently, various “omics” techniques were applied to identify the molecular changes and mechanisms of AF-associated remodeling. Proteomics and metabolomics were employed to investigate the entire proteome and metabolome levels in an indicated disease sample (Sühling et al., 2018; Chen et al., 2020). Both of them are complementary to genomics and transcriptomics, and are more sensitive to external factors and better reflect the real physiological status of a biological system. The rapidly-developed liquid chromatography—mass spectrometry (LC–MS)-based “omics” techniques achieve confirmed analysis of numerous metabolite or protein level patterns in biological samples, offering valuable data for biomarker screening and pathological and biological research (Lewis et al., 2008).

In this context, by integrating proteomics and metabolomics, we describe the dysregulated metabolites and proteins of AF patients relative to SR patients. Our results also provide candidate biomarkers for distinguishing valvular and non-valvular AFs from SR patients, reveal the fundamental differences between them and give a hint of emphasizing the available treatment strategies for AF.

## Methods

### Clinical Specimens

Left atrial appendages were harvested from AF patients undergoing cardiovascular surgery with procedures approved by the Ethics Committee Board of Shanghai East Hospital (0402017). Subjects (20 patients) suffered from long-standing persistent AF sustaining more than 1 year. Ten of them were recruited with rheumatic mitral stenosis, and the other ten were admitted after diagnosis with non-valvular AF. For comparison, ten matched SR patients of left atrial appendages were collected from healthy donors. The specimens were immediately snap-frozen and stored in liquid nitrogen.

### Partial Least Squares Discrimination Analysis (OPLS-DA)

The overall proteomic and metabolic changes in AF were determined by OPLS-DA on R package “mixOmics” (Rohart et al., 2017). Additionally, a univariate analysis of Wilcoxon Mann-Whitney U test was conducted for differential analysis.

### Differential Analysis and Pathway Enrichment

Dysregulated metabolites and protein were selected and mapped into the Kyoto Encyclopedia of Genes and Genomes (KEGG) database (http://home.jp/kegg/) for pathway enrichment analysis.

### Correlation Analysis Between Metabolomics and Proteomics

The correlative metabolites between metabolomics and proteomics were screened by Pearson correlation analysis. All the nodes were loaded to CytoScape for network construction in the light of the correlation data.

### Model Construction and Biomarker Identification

Receiver’s operating characteristic (ROC) curves were drawn with the R package “pRoC” for evaluating the diagnostic performance of metabolite biomarkers. We calculated the area under the curve (AUC) to assess the prediction accuracy.

### Statistical Analysis

We performed statistical analyses using R 3.6.2 and presented the data as mean ± standard error of mean (SEM). Grouped *t*-test was conducted for metabolomics and proteomics analyses between the AF and SR groups. The significant metabolites and proteins were determined according to *p* < 0.05 and foldchange (FC) ≥2; *p* < 0.05, respectively. KEGG enrichment analysis was conducted to assess the metabolic pathways. *χ* ^2^-test was applied for categorical variable analysis.

## Results

### Overview of This Workflow

**FIGURE 1:**
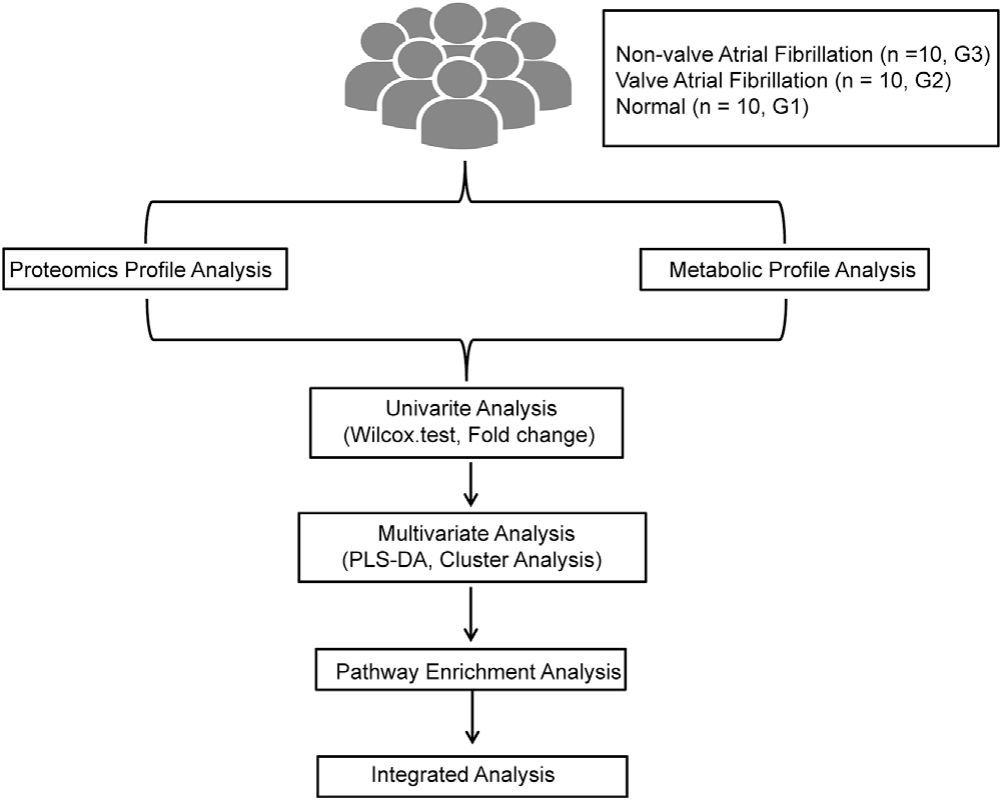
The flowchat of the analysis in this study.

Metabolomic and proteomic analyses were carried out using 10 persistent non-valvular AF samples, 10 valvular AF samples, and 10 SR samples. Through univariate and multivariate analyses, we analytically integrated the metabolic and proteomic data to find out significantly different metabolites and proteins between AF samples and SR samples. Further bioinformatic analyses were applied to reveal the significantly enriched pathways, providing clues about the pathological mechanism (Figure 1).

### Proteomics Profiling Analysis of Valvular AF and Non-Valvular AF Samples

**FIGURE 2:**
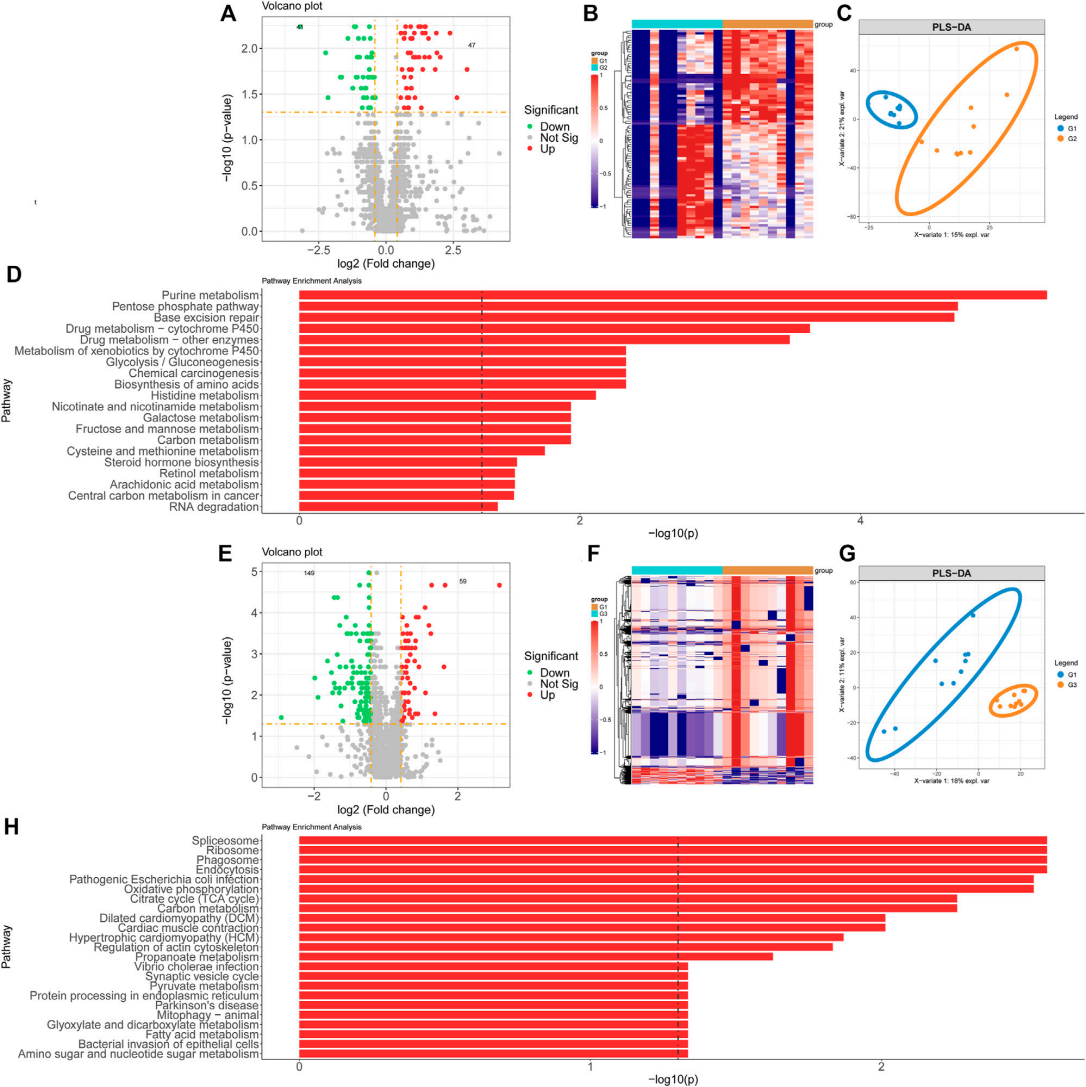
Proteomics profiling analysis of valvular AF and non-valvular AF samples. **(A, B)** The volcano plot **(A)** and heatmap **(B)** showed the differently expressed proteins in valvular AF compared to SR samples. **(C)** PLSDA analysis showed the protein profiles between valvular AF and SR tissues. **(D)** KEGG analysis of differently expressed proteins in valvular AF. **(E, F)** The volcano plot **(E)** and heatmap **(F)** showed the differently expressed proteins in non-valvular AF compared to SR samples. **(G)** PLSDA analysis showed the protein profiles between non-valvular AF and SR tissues **(H)** KEGG analysis of differently expressed proteins in non-valvular AF.

Proteomics samples were analyzed by univariate analysis. Differentially expressed proteins were identified with the criteria of false discovery rate (FDR)-adjusted *p* < 0.05 and FC > 2 for up-regulation, FDR-adjusted *p* < 0.05 and FC < 0.5 for down-regulation. There were 47 proteins with significant up-regulation and 41 proteins with significant down-regulation in valvular AF patients compared to the SR group. The dramatically up-regulated proteins in valvular AF were obviously down-regulated in the SR group (Figure 2B). Among them, both Integrin alpha -V heavy/light chain and Periodin were significantly changed, with the FCs of 0.108 and 7.941 respectively (Figure 2A). Additionally, PLS-DA of all protein expression data in these two groups found that the protein profile of valvular AF tissues was greatly distinguished from that of SR tissues, indicating that valvular AF underwent pathological alteration (Figure 2C). KEGG pathway enrichment analysis of differentially expressed proteins revealed totally 21 significantly expressed metabolic pathways, including multiple metabolic pathways such as Glycolysis/Gluconeogenesis, Metabolism of xenobiotics by cytochrome P450, Base excision repair, Pentose phosphate pathway, and Purine metabolism (Figure 2D).

Similar methods were applied to analyze the proteomics data of non-valvular AF, and found 59 significantly up-regulated proteins and 149 significantly down-regulated proteins in non-valvular AF compared to SR samples. The most significantly changed proteins included ATP-citrate synthase and Apolipoprotein E, with the FCs of 0.131 and 8.823, respectively (Figure 2E). Together with the analysis of valvular AF, PLSDA showed the protein profiles of non-valvular AF tissues were greatly distinguished from those of SR patient tissues, indicating non-valvular AF also underwent pathological alteration (Figures 2F,G). Then 24 significant pathways were predicted to be related to non-valvular AF, including Spliceosome, Ribosome, Phagosome, Endocytosis, Pathogenic *Escherichia coli* infection, Oxidative phosphorylation, Citrate cycle (TCA cycle), Dilated cardiomyopathy (DCM), and Cardiac muscle contraction. Interestingly, the enrichment pathways related to non-valvular AF were extremely different from those in valvular AF. For example, valvular AF was significantly related to Glycolysis/Gluconeogenesis, but non-valvular AF was more related to Citrate cycle (TCA cycle) (Figure 2H).

These analyses demonstrated that both valvular AF and persistent non-valvular AF tissues were greatly changed in protein levels compared with SR patient tissues and were strongly heterogeneous, implying the differences in the pathogenesis and clinical treatment should be distinguished between the two types of AF (Figure 2).

### Metabolomic Profiling Analysis of Valvular AF and Non-Valvular AF Samples

**FIGURE 3:**
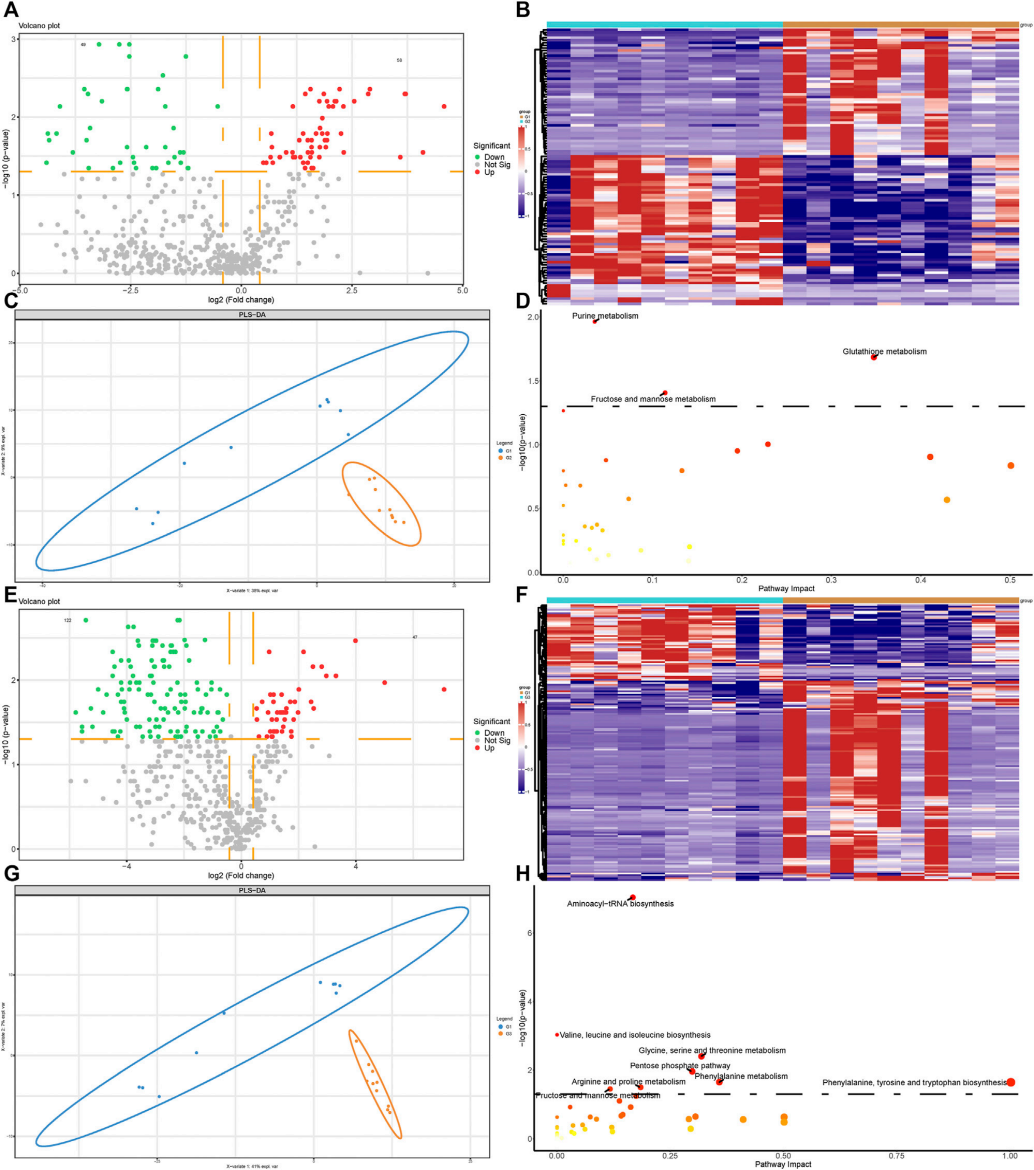
Metabolomic profiling analysis of valvular AF and non-valvular AF samples. **(A,B)** The volcano plot **(A)** and heatmap **(B)** showed the differently expressed metabolites in valvular AF compared to SR samples. **(C)** PLSDA analysis showed the protein profiles between valvular AF and SR tissues. **(D)** KEGG analysis of differently expressed metabolites in valvular AF. **(E,F)** The volcano plot **(E)** and heatmap **(F)** showed the differently expressed metabolites in non-valvular AF compared to SR samples. **(G)** PLSDA analysis showed the protein profiles between non-valvular AF and SR tissues **(H)** KEGG analysis of differently expressed metabolites in non-valvular AF.

We next performed metabolomics analysis of valvular AF and non-valvular AF samples. The differential analysis revealed 58 up-regulated and 49 down-regulated metabolites, both significantly, in valvular AF compared to the SR patient group (Figures 3A,B). Especially, the alterations of 20-hydroxy-PGF2a and Glutathione were the most significant, with FCs of 0.016 and 23.796, respectively. There were 47 up-regulated and 122 down-regulated metabolites, both significantly, in persistent non-valvular AF samples compared to SR patient groups (Figures 3E,F). Among them, Thymine and Thiopental were changed the most significantly, with FCs of 0.018 and 133.832, respectively. Besides, PLS-DA revealed that the metabolomics maps changed greatly in both valvular AF and persistent non-valvular AF compared to those from the SR patients, which are consistent with proteomics differences (Figures 3C,G). Notably, the signaling pathways of valvular AF and persistent AF were largely different in metabolomics enrichment analysis. The altered metabolites in valvular AF were mainly enriched in glutamine and fructose metabolism (Figure 3D), and those of non-valvular AF were significantly enriched in metabolism of valine, glycine, pentose, arginine, phenylalanine and fructose, and phenylalanine synthesis (Figure 3H). Fructose metabolism was the only common pathway between valvular AF and persistent AF (Figures 3D,H).

### Metabolomics and Proteomics Analyses of Valvular AF and Non-Valvular AF

**FIGURE 4:**
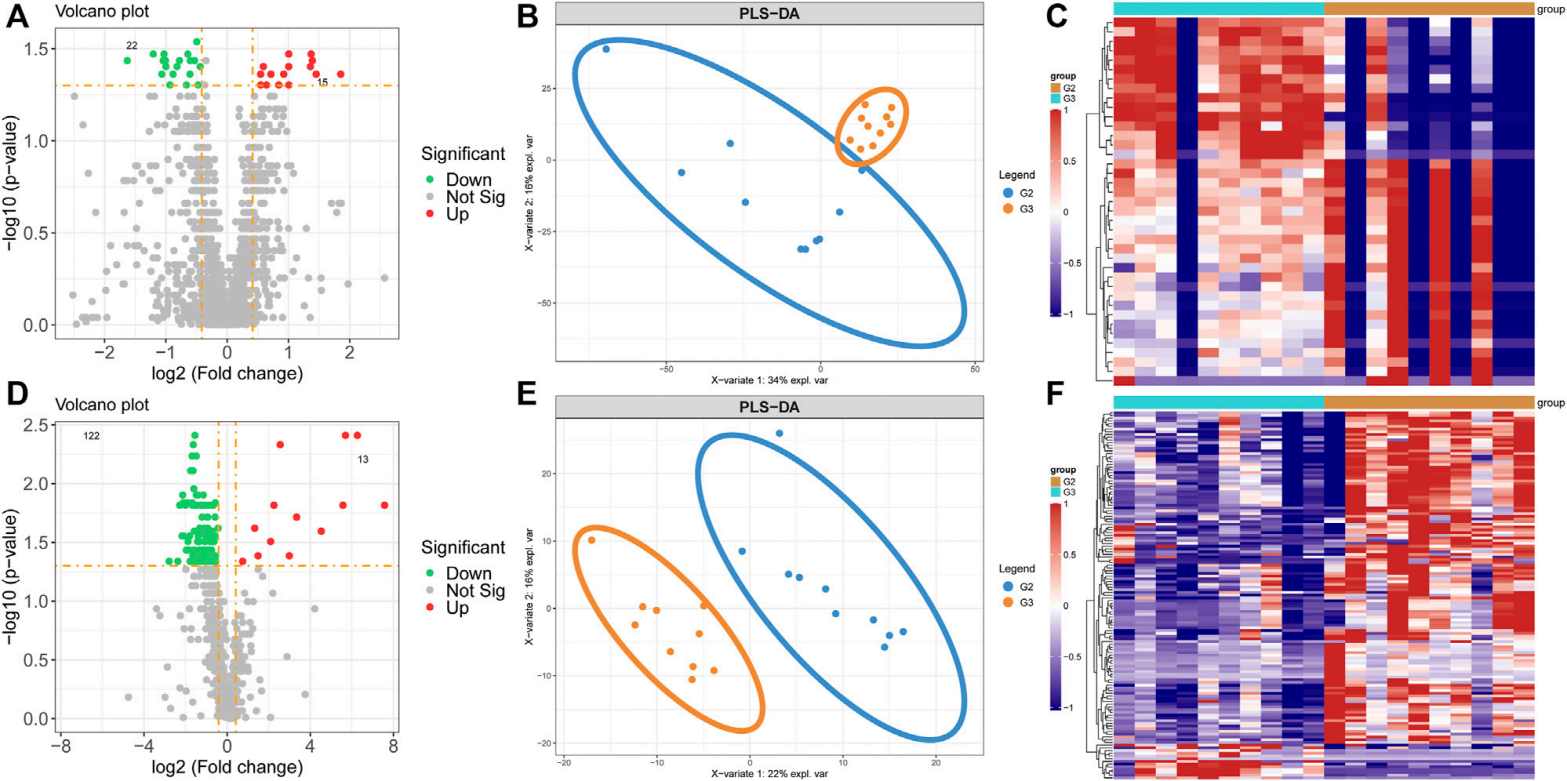
Metabolomics and proteomics analyses of valvular AF and non-valvular AF samples. **(A–C)** The volcano plot **(A)**, PLSDA analysis **(B)** and heatmap **(C)** showed the differently expressed proteins in non-valvular AF compared to valvular AF samples. **(D–F)** The volcano plot **(D)**, PLSDA analysis **(E)** and heatmap **(F)** showed the differently expressed metabolites in non-valvular AF compared to valvular AF samples.

By analyzing the differential levels of persistent non-valvular AF and valvular AF, we screened metabolites and proteins with FC > 1.5 and FC < 0.5 in the proteome and metabolome respectively compared to valvular AF (Figures 4A,D). There were 15 up-regulated proteins and 22 down-regulated proteins, 13 up-regulated metabolites and 122 down-regulated metabolites, all significantly, in persistent non-valvular AF compared to valvular AF samples. PLS-DA demonstrated that the protein and metabolism profiles of persistent non-valvular AF were not completely separated from those of valvular AF (Figure 4B), but can be fully divided by metabolomics analysis (Figure 4E). Hence, metabolomics differences suggest the pathological characteristics of persistent non-valvular AF and valvular AF, which exhibit higher similarity with phenotypic changes.

### Correlation Analysis of Differentially Expressed Proteins and Different Levels of Metabolites

**FIGURE 5:**
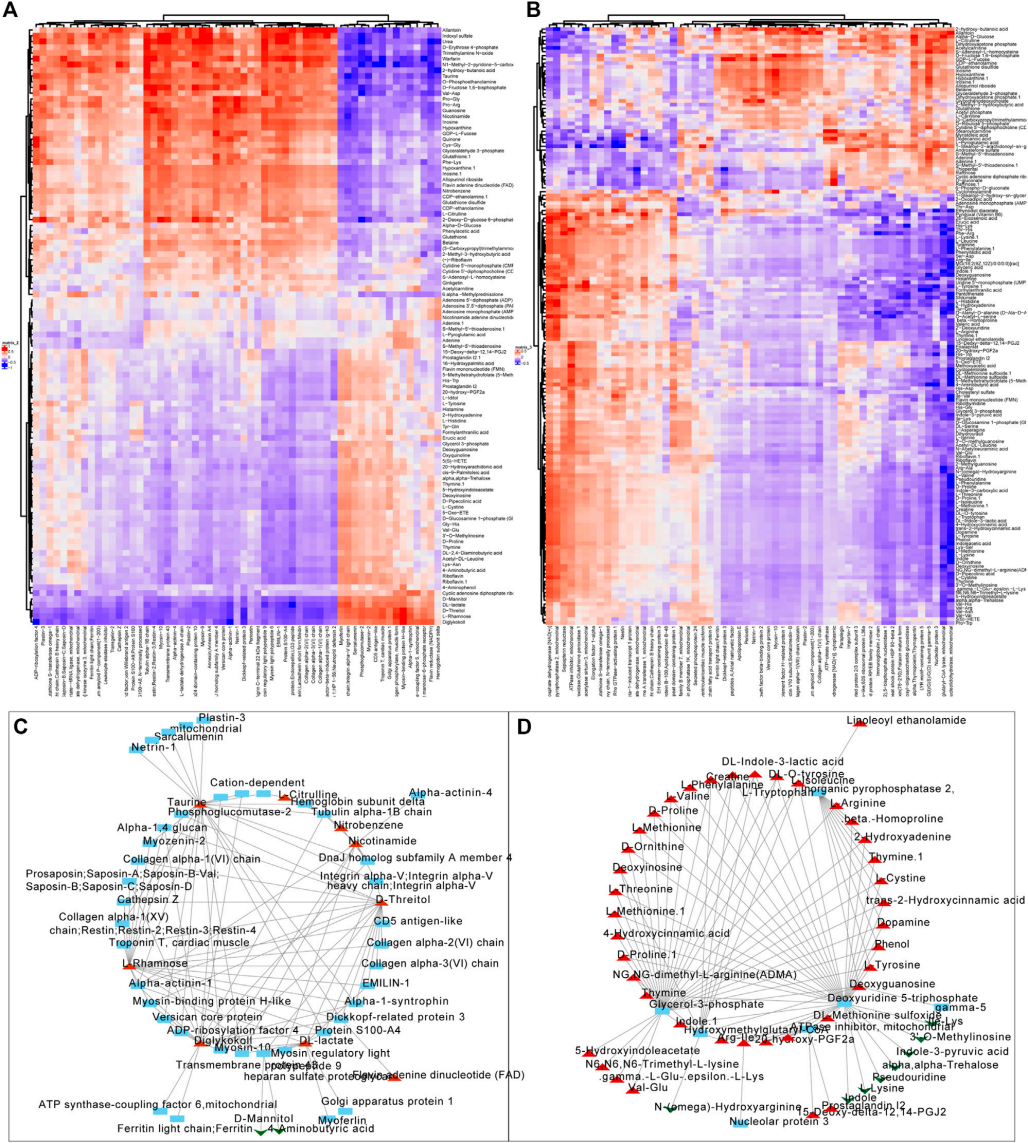
Correlation analysis of the differentially expressed proteins with metabolites. **(A,B)** The correlation analysis of differentially expressed proteins with metabolites in valvular AF **(A)** and non-valvular AF **(B)**. **(C, D)** We constructed differentially expressed proteins and metabolites interaction networks in valvular AF **(C)** and non-valvular AF **(D)**.

We firstly screened out the metabolites and proteins with FC > 1.5 and FC < 0.5 respectively in valvular AF and non-valvular AF tissues in comparison with the SR patient tissues. Among them, 104 metabolites and 59 proteins were found in valvular AF. Correlation analysis uncovered obvious clustering patterns in these proteins and metabolites, indicating the proteins and metabolites as-screened can represent the metabolism changes in valvular AF (Figure 5A). The 158 metabolites and 56 proteins in persistent non-valvular AF were screened, and had obvious clustering patterns according to correlation analysis, indicating these proteins and metabolites as-screened are representative of metabolic changes in persistent AF (Figure 5B). By sorting the absolute values of correlation coefficient, we drew a network diagram of the top100-related proteins and metabolites on Cytoscape. Several hub proteins with metabolites were identified in valvular AF and non-valvular AF. For example, Taurine, D-Threitol, L-Rhamnose, and DL-lactate all played crucial roles in valvular AF. Meanwhile, Glycerol-3-phosphate dehydrogenase, Inorganic pyrophosphatase 2, Hydroxymethylglutaryl-CoAlyase, and Deoxyuridine 5-triphosphate nucleotidohydrolase were crucial in non-valvular AF.

### Identification of Hub Networks to Predict Valvular AF and Non-Valvular AF

**FIGURE 6:**
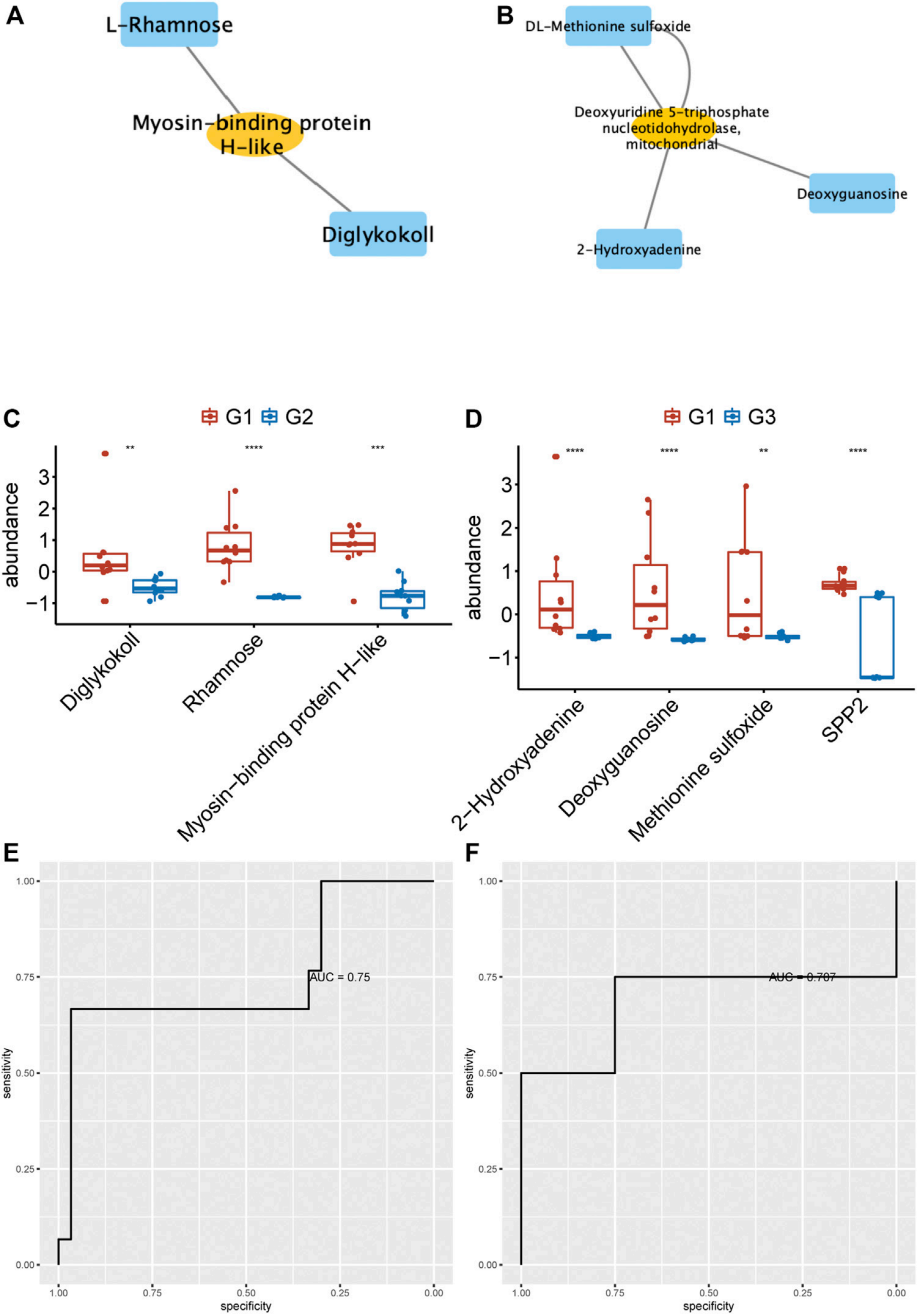
Identification of hub networks to predict valvular AF and non-valvular AF. **(A, B)** We identified the hub network in valvular AF **(A)** and non-valvular AF **(B)**. **(C, D)** The metabolites and proteins of hub networks were highly suppressed in valvular AF **(C)** and persistent non-valvular AF **(D)**.**(E, F)** The hub network can effectively distinguish valvular AF **(E)** and non-valvular persistent AF **(F)** from SR samples.

To identify the potential biomarkers to distinguish persistent non-valvular AF from valvular AF, we applied more stringent screening on proteomics and metabolomics. Then, the proteins-metabolites pairs with correlation coefficient > 0.6 were selected for further analysis. The hub networks in valvular AF included Diglykokoll, Rhamnose, and Myosin−binding protein H−like (Figure 6A), and the hub networks in non-valvular AF included 2−Hydroxyadenine, Deoxyguanosine, Methionine sulfoxide, and SPP2 (Figure 6B). These metabolites and proteins were highly suppressed in valvular AF and persistent non-valvular AF (Figures 6C,D). These substances were then applied to establish a model that can effectively distinguish valvular AF and non-valvular persistent AF from SR patient samples, with AUC of 0.75 (Figure 6E) and 0.707 (Figure 6F), respectively. Hence, these metabolites and proteins can be used as potential clinical molecular markers for discriminating two types of AF from SR patient samples.

## Discussion

AF is one of the most common and severe abnormal arrhythmias (Schmidt et al., 2011). Exploring ideal biomarkers for its diagnosis at early stage is still needed. Early detection of asymptomatic AF will offer a chance to take precautions against or to reduce undesirable disease consequences by implementing suitable treatment tactics. Over the past 20 years, catheter-based infrequent surgery and mixed ablation techniques are proven to be more successful in controlling heart rhythm of AF patients (Parkash et al., 2021). Unfortunately, the ablation technology efficiency varies largely among different clinical forms of AF, as it maximizes in paroxysmal AF and minimizes in long-term persistent AF (Margulescu and Mont, 2017). We comprehensively analyzed the dysregulated molecules in valvular AF and non-valvular AF samples and discovered significant differences in proteins and metabolites. The integrated multi-omics and bioinformatics data proved that identification of key pathways and characteristic genes in the two forms of AF and relevant AF-related metabolic pathways may help to study the underlying mechanism of AF and to unearth potential targets for diagnosis and treatment.

Proteomics is largely applied into research at large-scale protein levels, and enables researchers to investigate the protein alterations that lead to the pathological progression of diseases (Chambers et al., 2000; Sühling et al., 2018). Proteome can reflect the cell phenotype and variations that are potentially associated with cells and tissue functions (Mirauta et al., 2020). Compared with genetics, the proteomics and metabolomics are closely related to the phenotype of diseases and better manifest the disease progression (Barallobre-Barreiro et al., 2013). Over the past decades, several studies reveal the special metabolomics pattern in AF (Mayr et al., 2008; Jung et al., 2018). For example, Mayr et al. (2008) reported beta-hydroxybutyrate, ketogenic amino acid and glycine levels increased in persistent AF. Jung et al. (2018) found the levels of fatty acids and phospholipids were different between AF patients and SR patients. However, the special metabolomics patterns in valvular AF and non-valvular AF remain largely unknown. In this study, we metabolomically analyzed valvular AF and non-valvular AF samples to identify the potential roles of metabolomics in AF. The combined use of proteomics and metabolomics results revealed 47 up-regulated and 41 down-regulated proteins in valvular AF, and 59 up-regulated proteins and 149 down-regulated proteins in non-valvular AF compared to the SR patient samples. Meanwhile, we identified 58 up-regulated and 49 significantly down-regulated metabolites in valvular AF, and 47 up-regulated metabolites and 122 down-regulated metabolites in persistent non-valvular AF samples compared to the SR patient samples. KEGG pathway enrichment analysis revealed a total of 21 significantly enriched metabolic pathways, including multiple metabolic pathways such as Purine metabolism. Reportedly, plasma uric acid level increased in AF patients and was associated with AF burden. Interestingly, uric acid is an end product of purine metabolism (Guerreiro et al., 2009). Adenosine is purine metabolism-related metabolite that can induce AF. The purine metabolic pathway is closely implicated and strongly related to AF progression. For example, uric acid, the end-product of purine metabolism, plays a crucial role in cardiovascular diseases, such as atrial fibrillation and cardiovascular death. Increased uric acid levels in serum are related to AF (Watanabe, 2012; Borghi et al., 2020). Interestingly, the enrichment pathways related to non-valvular AF are extremely different from those in valvular AF. For example, valvular AF is significantly related to Glycolysis/Gluconeogenesis, but non-valvular AF is more related to Citrate cycle (TCA cycle). Reportedly, glycolysis activity was intensified in AF (Becerra-Tomás et al., 2021). Targeting Glycolysis may be a potential therapy for AF (Becerra-Tomás et al., 2021). Inducing the Warburg effect can markedly improve myocardial fibrosis remodeling in AF (Hu et al., 2019). However, the association between the glycolysis pathway and AF is unclear. We found Glycolysis played a crucial role in valvular AF, but not in non-valvular AF. Moreover, by comparing the differences inmetabolomics and proteomics between valvular AF and non-valvular AF samples, we found metabolomics differences illustrated the pathological characteristics of persistent non-valvular AF and valvular AF, exhibiting a higher similarity with phenotypic changes.

We next analyzed the correlations of differentially expressed proteins and metabolites. Several hub proteins with metabolites were identified in valvular AF and non-valvular AF. For example, Taurine, D-Threitol, L-Rhamnose, and DL-lactate played crucial roles in valvular AF. Meanwhile, Glycerol-3-phosphate dehydrogenase, Inorganic pyrophosphatase 2, Hydroxymethylglutaryl-CoAlyase, and Deoxyuridine 5-triphosphate nucleotidohydrolase were crucial in non-valvular AF. Interestingly, several of them were related to multiple crucial signaling in AF. For example, circulating glutamate and taurine levels are associated with the generation of reactive oxygen species in paroxysmal AF (Takano et al., 2016), and taurine prevents electrical remodeling in AF models (Yang et al., 2017). High-level lactate was observed in AF and related to AF remodeling, such as severe oxidative stress injury and mitochondrial control of apoptosis (Xu et al., 2013). We next identified two hub networks as potential biomarkers to distinguish persistent non-valvular AF and valvular AF from SR patient samples. The hub networks in valvular AF included Diglykokoll, Rhamnose, and Myosin−binding protein H−like. The hub networks in non-valvular AF included 2−Hydroxyadenine, Deoxyguanosine, Methionine sulfoxide, and SPP2. These models can effectively distinguish valvular AF and non-valvular persistent AF from SR patient samples, with AUCs of 0.75 and 0.707, respectively, indicating that these metabolites and proteins can be used as potential clinical molecular markers for discriminating two types of AF from SR patient samples.

## Conclusion

Through metabolomic and proteomic analyses, we identified differential levels of proteins and metabolites in non-valvular AF and valvular AF compared to SR samples, and found the huge metabolic profiling differences between non-valvular AF and valvular AF. KEGG pathway enrichment analysis showed altered proteins and metabolites were significantly related to metabolic pathways. Interestingly, the enrichment pathways related to non-valvular AF are significantly different from those in valvular AF. For example, valvular AF is significantly related to Glycolysis/Gluconeogenesis, but non-valvular AF is more related to Citrate cycle (TCA cycle). Based on correlation analysis of differential levels of proteins and metabolites, several hub proteins with metabolites were identified in valvular AF and non-valvular AF. For example, Taurine, D-Threitol, L-Rhamnose, and DL-lactate play a crucial role in valvular AF. Glycerol-3-phosphate dehydrogenase, Inorganic pyrophosphatase 2, Hydroxymethylglutaryl-CoAlyase, and Deoxyuridine 5-triphosphate nucleotidohydrolase are crucial in non-valvular AF. We next identified two hub networks as potential biomarkers, which can effectively distinguish valvular AF and non-valvular persistent AF from SR patient samples, with AUCs of 0.75 and 0.707, respectively. Hence, these metabolites and proteins can be used as potential clinical molecular markers to discriminate the two types of AF from SR patient samples. In summary, this study can provide a novel insight to understanding the mechanisms about the progression of AF and identifying novel biomarkers for the prognosis of non-valvular AF and valvular AF by using metabolomics and proteomics analyses.

## Data Availability

The original contributions presented in the study are included in the article/supplementary material, further inquiries can be directed to the corresponding authors.
